# Light and heavy chain deposition disease with focal amyloid deposition diagnosed with mass spectrometry: a case report

**DOI:** 10.1186/s12882-023-03207-0

**Published:** 2023-06-26

**Authors:** Yuki Shimamoto, Naoki Takahashi, Nagaaki Katoh, Yuki Matsui, Yusuke Mochizuki, Masanori Ito, Masahide Yazaki, Fuyuki Kametani, Kenji Kasuno, Yoshiki Sekijima, Hironobu Naiki, Masayuki Iwano

**Affiliations:** 1 Department of Nephrology, Faculty of Medical Sciences, University of Fukui, 23-3 Matsuokashimoaizuki Eiheiji-Cho, Yoshida-Gun, Fukui, Japan; 2 Department of Medicine (Neurology and Rheumatology), Shinshu University School of Medicine, Matsumoto, Nagano, Japan; 3 Department of Nephrology and Urology, Japanese Red Cross Fukui Hospital, Fukui, Japan; 4 Institute for Biomedical Sciences, Shinshu University, Matsumoto, Nagano, Japan; 5 Clinical Laboratory Sciences Division, Shinshu University Graduate School of Medicine, Matsumoto, Nagano, Japan; 6 Tokyo Metropolitan Institute of Medical Science, Setagaya-Ku, Tokyo, Japan; 7 Department of Pathology, University of Fukui, Fukui, Japan

**Keywords:** Light and heavy chain deposition disease, Amyloidosis, Mass spectrometry, Congo red, Birefringence, Nephrotic syndrome

## Abstract

**Background:**

Light and heavy chain deposition disease (LHCDD) is a rare condition characterised by the deposition of immunoglobulin components in the kidneys. Similarly, Amyloidosis is also caused by the deposition of light chain and/or heavy chain components of immunoglobulins which are folded into amyloid fibrils characterised by Congophilic deposits that exhibit apple-green birefringence under polarised light. Only a handful of reports describing LHCDD with amyloid fibril deposition have been previously published, however, none have characterized the composition of the deposited immunoglobulin components via mass spectrometry.

**Case presentation:**

We report a case of a 79-year-old Japanese woman with nephrotic syndrome. Bone marrow aspiration revealed a slight proliferation of plasma cells (under 10%). Immunofluorescence assessment of renal biopsy showed amyloid-like deposits in the glomerulus that were positive for IgA and kappa. Further, the Congo red staining of the deposits was faintly positive, and only a slight birefringence was detected. Electron microscopy confirmed fine fibrillar structures and non-amyloid deposits. Finally, mass spectrometry revealed that the deposits were composed of abundant amounts of light chain with small amounts of heavy chain. Therefore, the patient was diagnosed with LHCDD and focal amyloid deposition. Chemotherapy was subsequently initiated, which resulted in haematological and renal response. Under polarised light, faint birefringence with Congo red staining and periodic acid-methenamine silver positivity indicated that the deposits were mostly non-amyloid fibrils with a small component of amyloid fibrils. Generally, the diagnosis of heavy- and light-chain amyloidosis is defined by greater heavy chain deposition compared to the light chain. However, in our case, contrary to the definition, the light-chain deposition was far greater than that of the heavy-chain.

**Conclusions:**

This is the first case of LHCDD with focal amyloid deposition diagnosed by analysing the glomerular deposits by mass spectrometry.

**Supplementary Information:**

The online version contains supplementary material available at 10.1186/s12882-023-03207-0.

## Background

Light and heavy chain deposition disease (LHCDD) is a rare condition that was first reported in 1980 [1]. LHCDD is defined as the deposition of the light and heavy chain components of immunoglobulins, most frequently in the kidneys [2]. LHCDD is a subtype of non-amyloidotic monoclonal immunoglobulin deposition disease (MIDD), which includes light-chain deposition disease (LCDD), heavy-chain deposition disease (HCDD), and LHCDD. LCDD is the most prevalent MIDD, with a prevalence of 19% in patients with multiple myeloma [3]. In contrast, LHCDD is a rare subtype of MIDD.

Amyloidosis is characterised by the deposition of insoluble fibrils caused by abnormal protein folding. Immunoglobulin-related amyloidosis, the most common subtype of amyloidosis is characterised by the deposition of light- and/or heavy-chain immunoglobulins and various proteins such as serum amyloid P component and apolipoproteins [3]. Several organs, most frequently the kidneys, are affected by amyloidosis. Amyloidosis is diagnosed by visualising birefringence in Congo red staining and by the presence of unbranched amyloid fibrils measuring 5–15 nm in electron microscopy; however, it is sometimes difficult to distinguish amyloidosis from other deposition diseases.

Tandem mass spectrometry (MS) is a novel technique that is used to evaluate glomerular deposition. In this technique the glomeruli are microdissected from paraffin-embedded tissues using the laser capture technique. Peptides extracted from the glomeruli are then resolved by liquid chromatography MS. The results are then matched with a particular protein recorded in the database [4]. MS is an effective tool for assessing the components of glomerular deposits and has been utilized to accurately assess different deposition diseases. There have been a handful of reports on the simultaneous deposition of non-amyloids and amyloids [5–7], however, none have characterized the composition of the deposited immunoglobulin components via mass spectrometry. Therefore, here, we present the first case of LHCDD with focal amyloid deposition diagnosed by MS.

## Case presentation

### Clinical presentation and laboratory findings

A 79-year-old Japanese woman with a leg oedema visited her primary care physician, 3 months before being admitted to our hospital. Outpatient treatment with diuretics was initiated, but the oedema did not improve. Three days before admission, she experienced palpitations and fatigue, prompting her to again visit her primary care physician. Her laboratory data showed severe anaemia (haemoglobin [Hb], 58 g/L). Hence, she was subsequently admitted to our hospital.

**Fig. 1:**
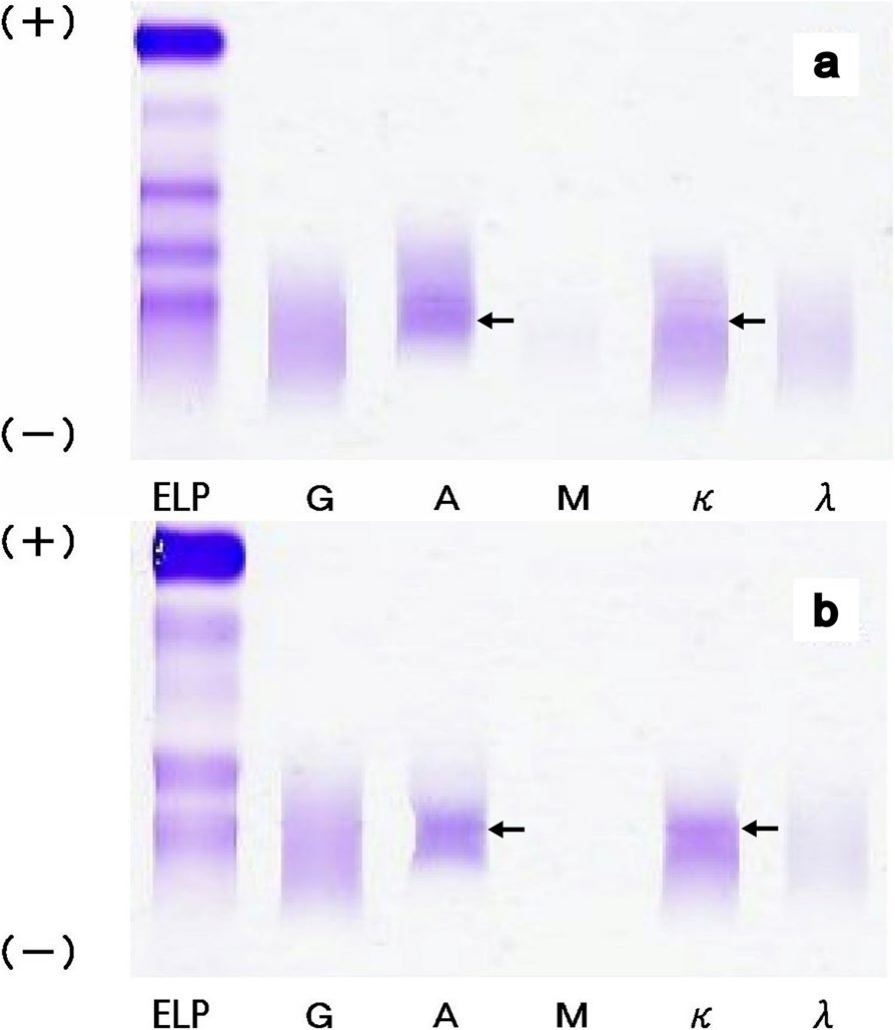
Serum and urinary immunoelectrophoresis (immunofixation method). Both serum (**a**) and urinary (**b**) immunoelectrophoresis showed IgA-kappa M protein (arrows)

She had no specific medical or any family history of renal disease. Physical examination revealed moderate leg oedema and kyphosis and no other indications of amyloidosis, such as numbness or signs consistent with polyneuropathy, gastrointestinal symptoms, macroglossia, orthostatic hypotension, purpura, or any changes to the skin. Laboratory data revealed microcytic anaemia (Hb, 50 g/L; mean corpuscular volume, 85.9 fL), hypoalbuminemia (albumin, 2.8 g/dL), and a possible slight decline in kidney function (serum creatinine, 0.64 mg/dL; estimated glomerular filtration rate, 66.9 mL/min/1.73 m^2^). Serum IgG, IgA, and IgM levels were 794 mg/dL, 1006 mg/dL, and 48 mg/dL, respectively. The serum free light chain (FLC) level for kappa and lambda was 77.4 mg/L and 15.2 mg/L, respectively, and the FLC ratio was 5.09. The brain natriuretic peptide level was 72.7 pg/mL. She also had iron and zinc deficiency. Serum and urine electrophoresis revealed the presence of IgA-kappa type M proteins (immunofixation method; Fig. 1). Serum β2-microglobulin was 4.6 mg/L. Bone marrow aspiration revealed a slight proliferation of plasma cells (6.8%). Urinalysis revealed an occult haematuria (1 +) and moderate proteinuria (1.34 g/gCr). Electrocardiography and cardiac ultrasound did not show any changes consistent with cardiomyopathy associated with amyloidosis, such as low voltage, thickening of the ventricular wall, or granular sparkling appearance. The chest radiograph revealed a slight bilateral pleural effusion, and the cardiothoracic rate was deemed to be 56.5%. For treating severe anaemia, red blood cell transfusion was administered in conjunction with iron and zinc supplementation, resulting in an improvement in anaemia (Hb 95 g/L) and her subsequent discharge. After discharge, her urinary protein increased to 5.7 g/gCr, and serum albumin decreased to 2.8 g/dL. The patient was then diagnosed with nephrotic syndrome and a renal biopsy was performed.

### Renal biopsy

**Fig. 2:**
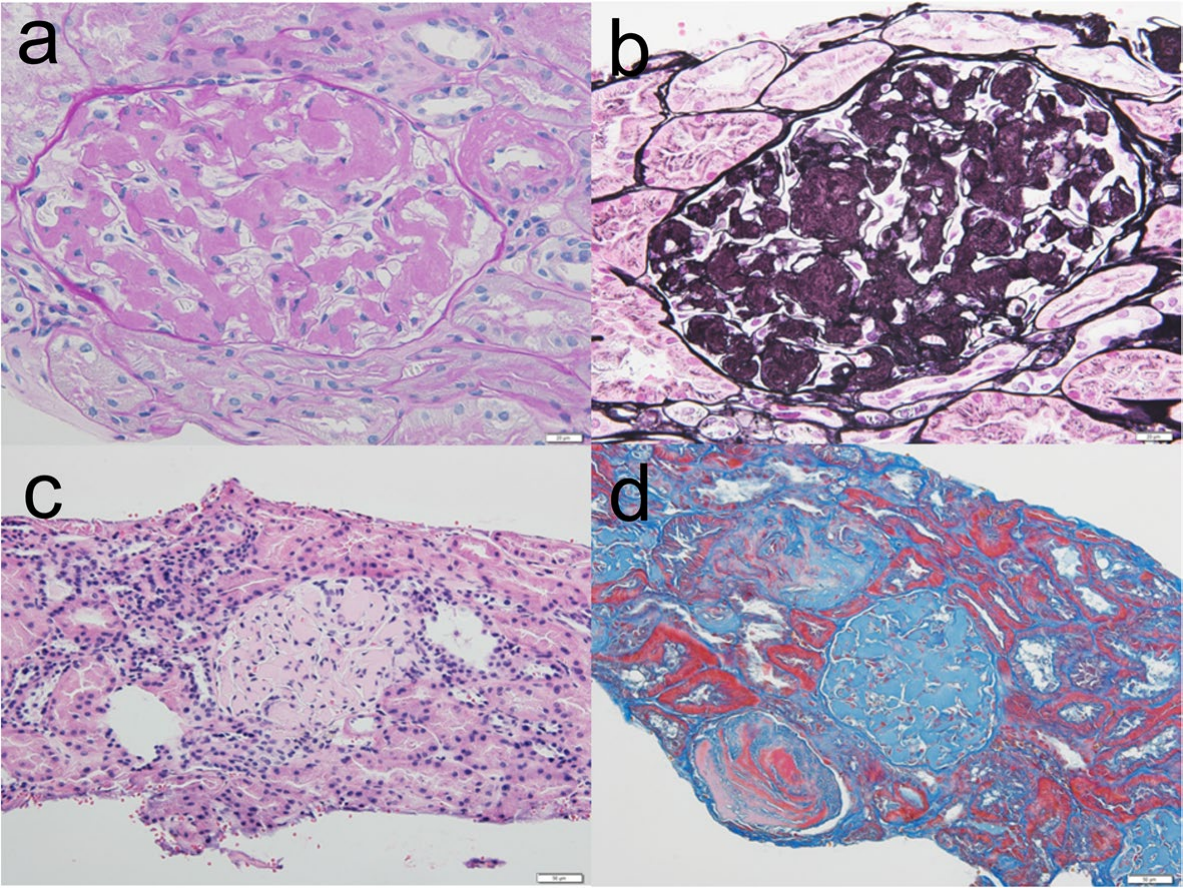
Accumulation of deposits in the glomeruli. Renal biopsy revealed accumulation of abundant eosinophilic and strongly methenamine-positive deposits in the glomeruli with **a** PAS staining (× 400), **b** PAM staining, **c** haematoxylin and eosin staining (× 200), and **d** Masson’s trichrome staining (× 200)

**Fig. 3:**
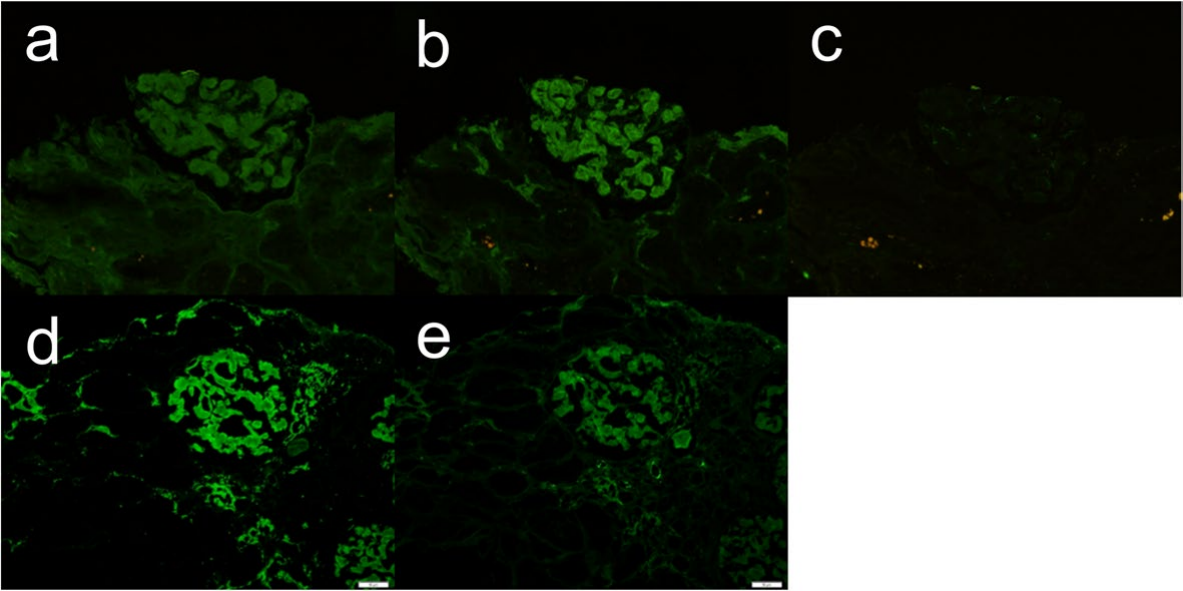
IgA- and kappa-positive staining by immunofluorescence stain. Immunofluorescence staining revealed positive IgA and kappa with **a** IgG (× 200), **b** IgA (× 200), **c** IgM (× 200), **d** kappa (× 200), and **e** lambda (× 200)

**Fig. 4:**
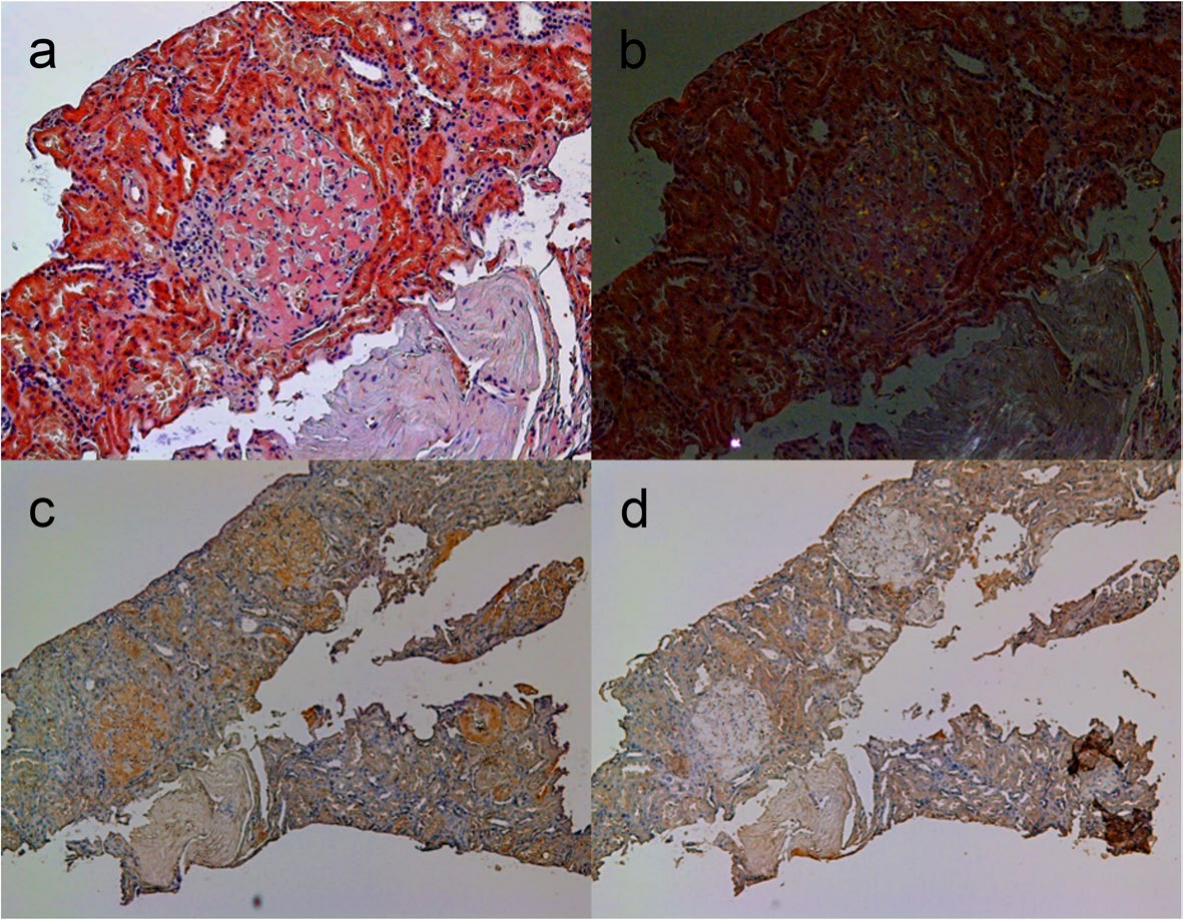
Congo red and immunohistochemical staining. Glomeruli are **a** faintly Congo red-positive (× 100) and **b** have faint birefringence under polarised light (× 100). Immunohistochemical staining showed kappa positive, **c** kappa (× 50), **d** lambda (× 50)

**Fig. 5:**
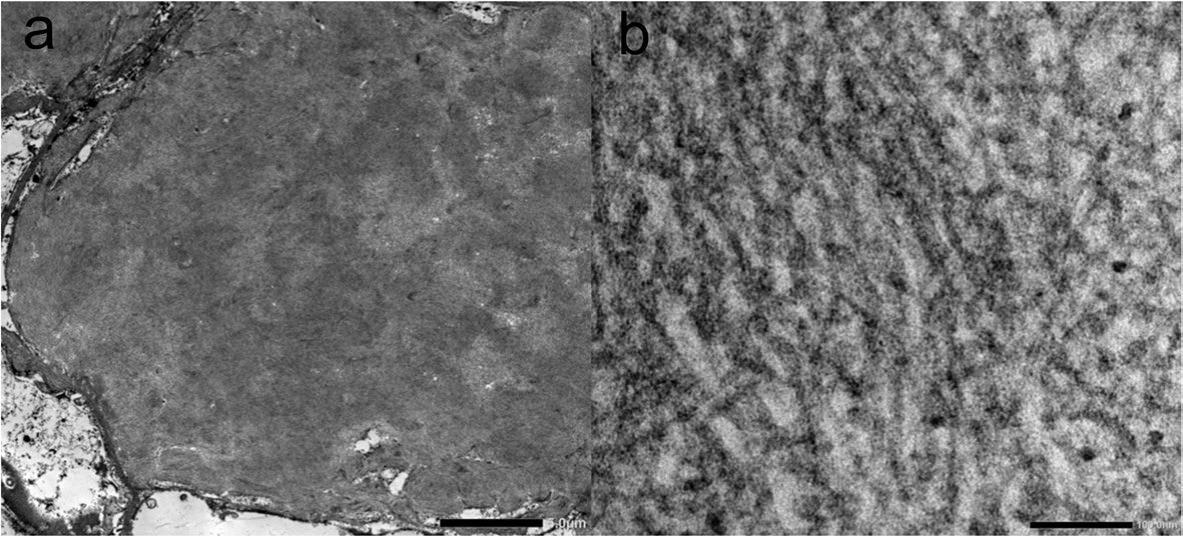
Fibrillary deposits detected by electron microscopy. Electron microscopy of the formalin-fixed sample revealed unbranched fibrils measuring 10–15 nm **a** (× 1,200) and **b** (× 60,000)

Light microscopy revealed methenamine-positive deposits in the mesangial and paramesangial regions (Fig. 2). Some glomeruli were accompanied by segmental sclerosis caused by the deposits. Interstitial fibrosis and tubular atrophy were mild. Immunofluorescence staining was positive for IgA and kappa chains in the expanded mesangial area (Fig. 3). The tubular basement membrane was partly granular and positive for kappa chains. Additional Congo red staining showed mild positivity in the glomerular deposits, but only faint birefringence. Immunohistochemical staining of the kappa chains was mildly positive (Fig. 4). Furthermore, electron microscopy was conducted with formalin-fixed specimen to evaluate glomerular deposits, which revealed unbranched fibrils in glomerular deposits measuring 10–15 nm (Fig. 5).

### Laser microdissection (LMD)–liquid chromatography-tandem mass spectrometry (LC–MS/MS)

**Fig. 6:**
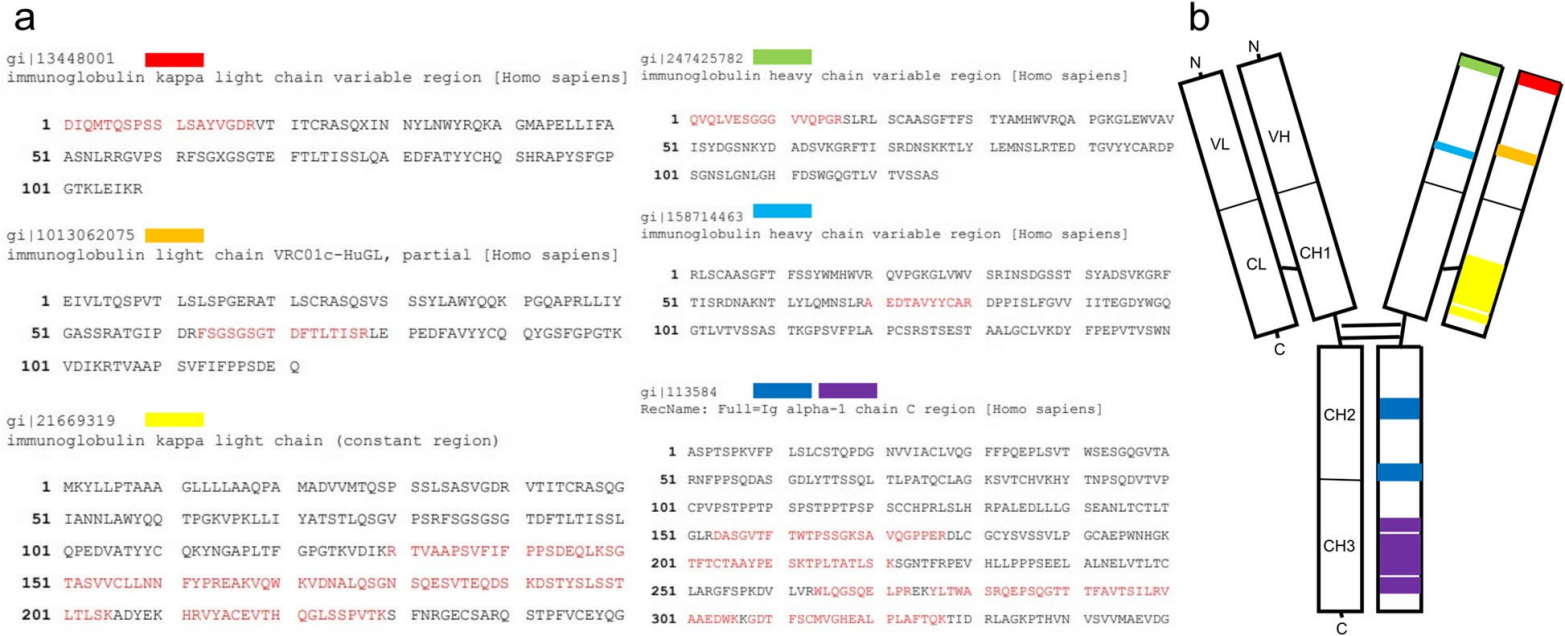
Detailed account of peptides detected by MS. The highlighted red letters show the actual peptides detected by the MS (**a**), and pictorial presentation of the detected part of immunoglobulin molecule (**b**)

LMD-LC–MS/MS revealed that the deposits consisted significant amounts of the kappa constant region of immunoglobulin. Additionally, a small amount of the alpha-1 constant region was also detected along with amyloid-associated proteins (Table 1). Details of the detected protein and an illustration of globulin are presented in Fig. 6. The emPAI (exponentially modified protein abundance index) is often used as an index for estimating protein abundance during proteomic analyses using mass-spectrometry. The emPAI value represents the relative amount of each protein contained within the sample [8]. Therefore, a higher value emPAI is indicative of a larger amount of protein, compared to the other proteins in the sample.

**Table 1:** Liquid chromatography-tandem mass spectrometry (LC–MS/MS) assessment

Description	emPAI
Immunoglobulin kappa constant OS=Homo sapiens OX=9606 GN=IGKC PE=1 SV=2	**37.99**
Apolipoprotein E OS=Homo sapiens OX=9606 GN=APOE PE=1 SV=1	**12**
Vitronectin OS=Homo sapiens OX=9606 GN=VTN PE=1 SV=1	**1.72**
Clusterin OS=Homo sapiens OX=9606 GN=CLU PE=1 SV=1	**1.23**
Immunoglobulin heavy constant alpha 1 OS=Homo sapiens OX=9606 GN=IGHA1 PE=1 SV=2	**1.22**
Serum amyloid P-component OS=Homo sapiens OX=9606 GN=APCS PE=1 SV=2	**1.02**
Apolipoprotein A-I OS=Homo sapiens OX=9606 GN=APOA1 PE=1 SV=1	**0.97**
Apolipoprotein A-IV OS=Homo sapiens OX=9606 GN=APOA4 PE=1 SV=3	**0.22**
Basement membrane-specific heparan sulphate proteoglycan core protein OS=Homo sapiens OX=9606 GN=HSPG2 PE=1 SV=4	**0.14**

LC–MS/MS revealed abundant kappa chains, minimal amounts of IgA heavy chains, and amyloid-associated proteins

*Abbreviation*: *emPAI* Exponentially modified Protein Abundance Index

### Clinical course

Based on the above findings, the patient was diagnosed with LHCDD and focal amyloid deposition. Skin biopsy did not show amyloid deposition, while gastrointestinal biopsy revealed faint birefringence, suggesting that the patient had systemic amyloidosis. Bone marrow aspiration was repeated, which revealed an increase in the plasma cell population (14.4%). The increase in plasma cell population and a concurrent anaemia indicated multiple myeloma [9]. She did not however present any other myeloma-defining events, such as hypercalcemia, decline in renal function, and bone legions. We started cyclophosphamide 300 mg/m^2^/day, bortezomib 1.3 mg/m^2^, and dexamethasone treatment 20 mg/week followed by weekly administration of daratumumab (1, 800 mg/week). After 9 months of treatment, proteinuria improved from 8.0 to approximately 5.0 g/gCr in the absence of decrease in eGFR, indicating a renal response [10]. Serum albumin concentration also gradually increased, leading to a reduction in leg oedema. The reduction in serum M-protein (IgA 467 mg/dL) and the difference between involved and uninvolved FLC (dFLC; 20.8 mg/L) was greater than 50%, but less than 90% (haematological partial response [11]).

## Discussion and conclusions

In this study, we report an exceptional case of IgA–kappa-type LHCDD accompanied by focal amyloid deposition. Cases of MIDD with amyloid deposition have previously been reported [5–7]; however, these reports did not provide a detailed characterization of the deposits. Additionally, MS was not primarily employed to diagnose these cases. Therefore, this is the first case of LHCDD with focal amyloid deposition that was diagnosed by MS analyses of the glomerular deposits.

Several factors in the present case suggest non-amyloid-like features of the deposits.

First, the deposits were only slightly Congophilic. As the birefringence was initially extremely weak to be observed, higher magnification was required, which revealed a faint birefringence under polarised light. However, the intensity of birefringence appeared to be weaker than those reported in the previous cases [3, 7]. Birefringence occurs due to the unique beta-pleated sheet structure of amyloid fibrils. The glomerular deposits in our case showed faint birefringence mainly in the periphery, in contrast to the previous cases where the birefringence was observed primarily in the mesangial and capillary deposits. The accumulation of numerous light chains in the glomeruli, in our case, may not have formed the beta-pleated sheet structure, characteristic of the amyloids.

Furthermore, these fibrillar deposits may not form typical amyloid fibrils. Based on previous cases of LCDD with fibrillar deposits and negative Congo red staining, our findings suggest that the fibrillar deposits originated from immunoglobulin light-chain fragments but did not form a typical tertiary amyloid structure [12–14]. A previous case also reported simultaneous deposition of fibrillar structures and powdery deposits in the glomerulus [12]. Similarly, another case report reported the presence of amyloid and non-amyloid fibrillar deposition in the glomerulus [5]. Although the sample in our case was formalin-fixed, fibrillar structures under high magnification during electron microscopy were still observed, which might have been composed of amyloid and non-amyloids.

Second, the deposits were strongly stained with periodic acid-Schiff (PAS) and periodic acid-methenamine silver (PAM). In cases of renal amyloidosis, amyloid deposits are weakly positive with PAS staining and argyrophilia is lost due to the expanded mass of amyloid deposits that replace the normal mesangial matrix [15]. In contrast, MIDD deposits are strongly argyrophilic due to the expanded mesangial matrix, accompanied by the immunoglobulin component and extracellular matrix proteins [12, 15]. Additionally, it has been indicated that transforming growth factor (TGF)-β plays a key role in the production of mesangial matrix protein in LCDD [16]. Therefore, strong positivity with PAS and PAM suggests that the deposits in our case were not typical amyloids.

Initially, we suspected our case to be one of immunoglobulin heavy- and light-chain (AHL) amyloidosis. However, it did not fit the characteristics for the diagnosis of AHL which is characterised by (i) equal immunofluorescence staining for both heavy and light chains or (ii) large amount of both heavy and light chain detection by LMD/MS [17]. Furthermore, previous cases of AHL amyloidosis had greater deposits of heavy chains than that of light chains [17, 18]. In our case, there was an abundance of light chains, whereas the amount of heavy chains was minimal; these features were not consistent with those of AHL amyloidosis.

The co-deposition of amyloid fibrils and non-amyloid immunoglobulins is a well-described phenomenon [19–21]. Nevertheless, there are few reports of amyloid and non-amyloid co-deposition evaluated by MS. Manabe et al. attempted to distinguish amyloidogenic and non-amyloidogenic fibrillar deposits using MS [5]. They performed amyloid purification followed by MS, revealing co-deposition of immunoglobulin light-chain (AL) amyloid and non-amyloid heavy chains. Their case had the deposition of predominantly AL amyloids and the simultaneous deposition of non-amyloid monoclonal immunoglobulins. In addition, they raised a question about the simultaneous development of AL and AH amyloidosis, due to the extremely rare prevalence of AH amyloidosis. MS findings in our case were in obvious contrast to those of AHL amyloidosis [17]. Therefore, we believe that our case did not have AHL amyloidosis but a simultaneous deposition of non-amyloid and amyloid deposits. Although AHL amyloidosis have been diagnosed by the intensity of immunofluorescence staining or the number of MS deposits so far [17], these diagnostic tools do not demonstrate that all these deposits form amyloid fibrils. The present case therefore suggests that MS, combined with other diagnostic tools, may be an effective tool to distinguish between cases of amyloid and non-amyloid co-deposition from actual AHL amyloid cases.

In contrast to a case of AL amyloidosis with the deposition of non-amyloid monoclonal immunoglobulins [5], our case showed IgA–kappa-type LHCDD with focal amyloid deposition. The kappa chain was positive with immunohistochemical staining. MS revealed large deposits of the kappa constant region, whereas only small amounts of heavy-chain components were detected. However, simultaneous deposition of heavy chains was observed on the basis of positive IgA immunofluorescence staining. Slight positivity with Congo red staining suggests that most of the kappa chains did not form the unique tertiary structure of amyloids. Therefore, we diagnosed the patient as having IgA–kappa-type LHCDD with focal amyloids. MS does not provide the information on three-dimensional structure of the proteins, which is one of the limitations of this tool. In our present case, MS plays an additional role in the evaluation of deposits. However, histological findings from other tools, combined with MS data, have led us to the most likely diagnosis in this case, wherein immunoglobulin and amyloid fibril deposition occurred simultaneously. The future accumulation of MS data from similar cases may help clarify the mechanisms of amyloid fibrillation.

We report a case of LHCDD with focal amyloids. MS may be an effective tool that can help characterise glomerular deposits.

## Supplementary Information

**Additional file 1: Supplementary Figure.** Serum and urinary immunoelectrophoresis (immunofixation method) with several contrast from -20% to +20%.

## Data Availability

Anonymized data can be provided for reasonable request. Contact corresponding author: yk-shima@u-fukui.ac.jp.

## Abbreviations

LC–MS/MS: Liquid chromatography–tandem mass spectrometry
LMD: Laser microdissection;
LCDD: Light chain deposition disease
HCDD: Heavy chain deposition disease
LHCDD: Light and heavy chain deposition disease
AL amyloidosis: Immunoglobulin light-chain amyloidosis
AH amyloidosis: Immunoglobulin heavy-chain amyloidosis
AHL amyloidosis: Immunoglobulin heavy- and light-chain amyloidosis
