# Supplementary figures and images for: Light and heavy chain deposition disease with focal amyloid deposition diagnosed with mass spectrometry: a case report
Source: BMC Nephrol. 2023 Jun 26;24:187. doi: 10.1186/s12882-023-03207-0 (PMC10294544; doi:10.1186/s12882-023-03207-0)

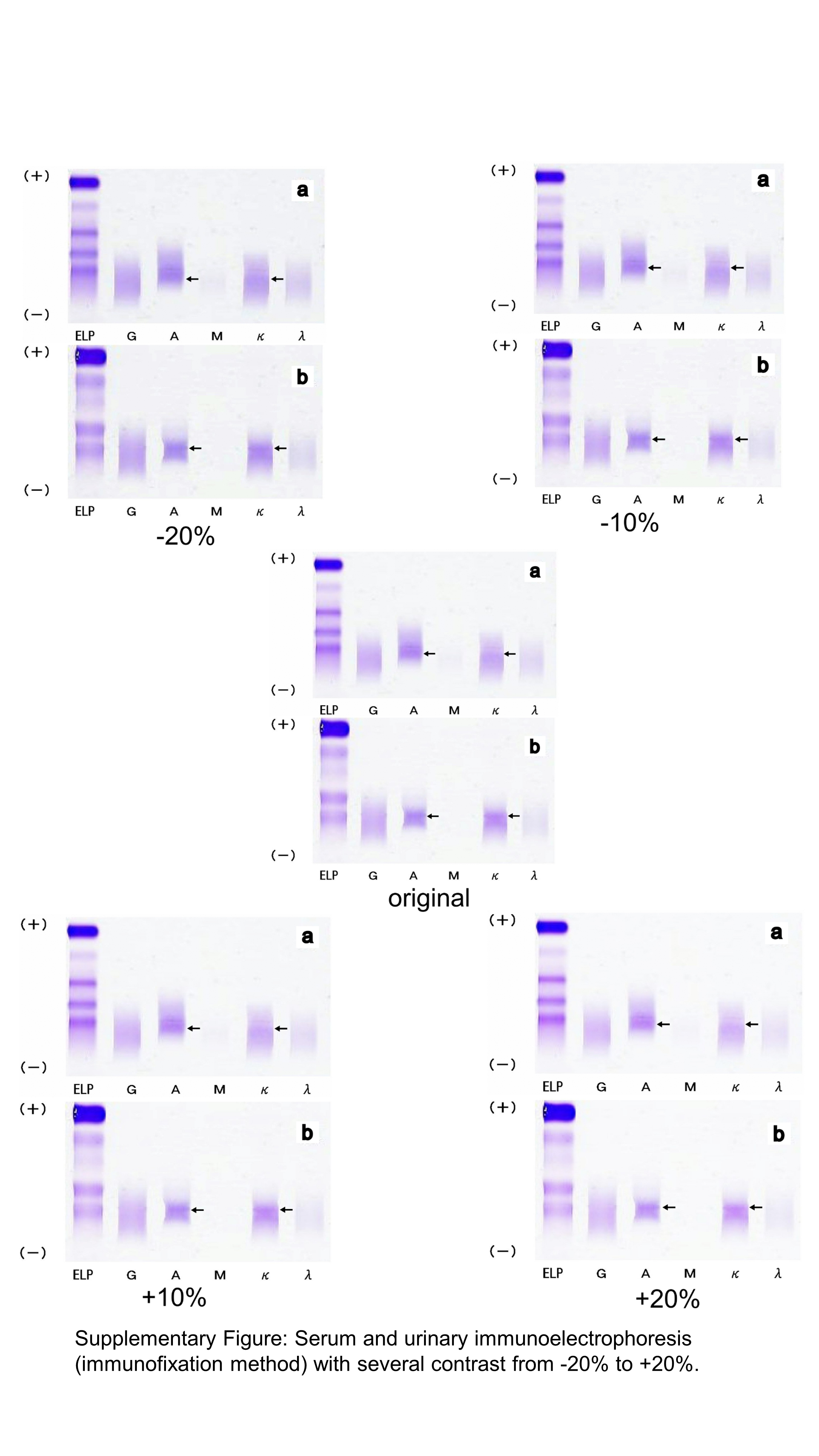

Supplement: Supplementary file 1 — Additional file 1: Supplementary Figure. Serum and urinary immunoelectrophoresis (immunofixation method) with several contrast from -20% to +20%. [file 12882_2023_3207_MOESM1_ESM.png]
